# Evaluating the quality of ChatGPT-generated medical information on major ophthalmic conditions: A comparative assessment against the EQIP tool and guidelines

**DOI:** 10.1371/journal.pone.0334250

**Published:** 2025-10-16

**Authors:** Mingfang Hu, Pingping Zou, Teng Li, Yuying Wang

**Affiliations:** Ophthalmology Department, The First Affiliated Hospital of Zhengzhou University, Zhengzhou, China; Children’s National Hospital, George Washington University, UNITED STATES OF AMERICA

## Abstract

**Background:** The use of artificial intelligence for creating medical information is on the rise. Nonetheless, the accuracy and reliability of such information require thorough assessment. As a language model capable of generating text, ChatGPT needs a detailed examination of its effectiveness in the healthcare domain.

**Objective:** This research sought to evaluate the precision of medical data produced by ChatGPT-4o (https://chat.openai.com/chat, accessed Mar. 12, 2025), concentrating on its capability to handle the top five ophthalmic issues that pose the greatest global health challenges. Furthermore, the investigation compared the AI’s answers to recognized medical guides.

**Methods:** This research employed an adapted version of the Ensuring Quality of Information for Patients (EQIP) instrument to evaluate the quality of ChatGPT’s replies. The guidelines for the five conditions were rephrased into pertinent queries. These queries were then fed into ChatGPT, employing benchmarking against established ophthalmology clinical guidelines, and the resulting answers were independently scrutinized for precision and consistency by two investigators. The consistency among raters was evaluated using Cohen’s kappa value.

**Results:** The median EQIP score across the five conditions was 18 (IQR 18-19). The modified EQIP instrument revealed a robust consensus between the two evaluators when assessing ChatGPT’s responses, as indicated by a Cohen’s kappa value of 0.926 (95% CI 0.875-0.977, P<0.001). The alignment between the ChatGPT responses and the guideline recommendations was 84% (21/25), as indicated by a Cohen’s kappa value of 0.658 (95% CI 0.317-0.999, P<0.001).

**Conclusions:** ChatGPT demonstrates robust quality and guideline compliance in producing medical content. Nevertheless, improvements are necessary to enhance the accuracy of quantitative data and ensure a more comprehensive coverage, thereby offering valuable insights for the advancement of medical information generation.

## 1 Introduction

The rapid evolution of technology continues to transform healthcare delivery, as evidenced by advancements in engineered nanomaterials [1] and point-of-care diagnostic technologies [2]. Generative Language Models (GLMs) demonstrate advanced natural language processing capabilities, with emerging applications across medical domains including medical licensing examinations [3], patient education [4], and medical diagnosis [5], potentially supplementing traditional medical information sources. The potential shift toward AI-generated medical advice warrants careful evaluation against established clinical knowledge bases. The reliability of such information requires rigorous assessment. In healthcare contexts where erroneous information may have serious consequences [6], GLMs demand substantially greater scrutiny than applications in non-clinical domains. This is particularly relevant to specialized fields like ophthalmology, where comprehensive evaluation is needed to establish GLMs’ capacity for clinically consequential applications.

Despite being the most recent version of GLMs, ChatGPT’s capability to produce medical data for severe ophthalmic conditions has not been comprehensively assessed. This research sought to assess the accuracy of data produced by ChatGPT regarding ophthalmic disorders and examine how well its responses align with established ophthalmology standards guidelines. The results will offer valuable perspectives on the possible clinical uses of the model in ophthalmic care, while also pinpointing aspects that need enhancement. This will aid in the development of more accurate medical AI technologies.

## 2 Methods

### 2.1 ChatGPT version and selection of medical conditions

This research employed OpenAI’s ChatGPT-4o, launched on May 13, 2024 [7], to gather data. Utilizing the Global Burden of Disease database, the study focused on five ophthalmic conditions that carry the most significant global health impact for analysis: cataracts, refractive errors, glaucoma, age-related macular degeneration (AMD), and diabetic retinopathy (DR) [8–10].

### 2.2 Modified EQIP instrument and data entry

#### 2.2.1 Analysis of modified EIP instrument.

This research employed an adapted version of EQIP instrument for assessment. The EQIP tool is a validated and reproducible means of evaluating written patient information, highlighting its capabilities in providing a robust assessment of online patient resources. The adjusted EQIP instrument includes 36 components grouped into three sections: content (Items 1-18), publication or revision date (Items 19-24), and structural information (Items 25-36). The content domain reviews whether sufficient medical information is incorporated in the resource. The publication or revision date domain looks at the degree to which the website displays production details. The structure domain analyses the readability and construction of the resource [11]. This tool was utilized to assess five conditions. Every element of the revised EQIP instrument was reworded into a query and then presented to ChatGPT. The prompts were deliberately unambiguous and minimally embellished. For instance, regarding cataracts, the instruction for Item 3, “Description of the medical problem/treatment/procedure”, was standardized as: “Describe the medical problem of cataracts”. Item 4, “Definition of the purpose of the interventions”, was standardized as: “What is the purpose of medical interventions in cataracts”. To maintain result consistency, each query was entered three separate times. The produced answers were then synthesized and compiled. Responses were labeled “1” for accurate and comprehensive, “0” for inaccurate, incomplete, or inconsistent, “N/A” for non-applicable. “0 ”: AI hallucinations, responses that are entirely unrelated to the context or are complete fabrications. Two researchers (both are ophthalmology specialists with over 20 years of clinical experience, holding the senior title of Chief Physician) independently assessed all the responses, and any disagreements in their evaluations were resolved through collaborative discussions.

#### 2.2.2 Analysis of guideline agreement.

This research assessed the consistency of ChatGPT’s answers in accordance with established ophthalmology guidelines. Guidelines provided by the UK National Institute for Health and Care Excellence of cataracts, glaucoma, AMD, and DR have been used as benchmarks for alignment analysis [12–15]. For refractive errors, where no relevant guidelines are available from NICE, guidelines from the American Academy of Ophthalmology were adopted for comparison [16]. During this procedure, five distinct suggestions from every guideline, covering disease identification, risk elements, and therapeutic strategies, were rephrased into focused inquiries and then fed into the ChatGPT. The researchers generated, recorded, and methodically compared ChatGPT’s responses to each question against the relevant guideline recommendations. Responses that matched the guidelines were assigned a score of “1,” whereas those that diverged from the guidelines were given a score of “0.”

### 2.3 Statistical analysis

Data processing and analysis were performed using R (latest version), along with Zstats v1.0 (www.zstats.net). In the statistical evaluation, all data points were presented as medians (with interquartile ranges [IQR]) or as counts (with percentages).

This study does not involve any patient interventions and therefore does not require ethics approval or informed consent.

## 3 Results

### 3.1 Content, publication or revision date, and structural data of EQIP instrument

The modified EQIP instrument now caps at a total of 36 points, segmented into three parts: content, publication or revision date, and structural data. The content segment can earn up to 18 points, focusing on the thoroughness and precision of the data provided. The section for the publication or revision date can earn up to 6 points, highlighting the importance of clear and identifiable information sources. The structural data segment can earn up to 12 points, evaluating the level of information organization and its practical utility. Across the five conditions examined, the modified EQIP’s median overall score was 18 (IQR 18-19), and the score distributions were uniform for all conditions. The specifics can be found in Table 1.

**Table 1:** Summarized findings evaluated with the adapted EQIP instrument.

Item	Cataracts (18)	Refractive errors (18)	Glaucoma (19)	AMD (18)	DR (20)
1. Initial definition of which subjects will be covered	N/A	N/A	N/A	N/A	N/A
2. Coverage of the previously defined subjects	N/A	N/A	N/A	N/A	N/A
3. Description of the medical problem or treatment/procedure	1	1	1	1	1
4. Definition of the purpose of the interventions	1	1	1	1	1
5. Description of treatment alternatives	0	0	1	0	0
6. Sequence of interventions / surgical procedure	0	0	0	0	1
7. Qualitative benefits for the patient	1	1	0	0	1
8. Quantitative benefits to the patient	0	0	0	0	0
9. Qualitative risks and complications	1	0	1	1	1
10. Quantitative risks and complications	0	0	0	0	0
11. Addressing quality-of-life issues	1	1	1	1	1
12. Handling of complications	1	1	0	1	0
13. Patient precautions	1	1	0	1	0
14. Mention of alert signs	1	1	1	1	1
15. Costs and insurance issues	0	0	0	0	0
16. Contact details for hospital services	N/A	N/A	N/A	N/A	N/A
17. Other reliable sources of info/support	1	0	1	1	1
18. Coverage of all relevant issues	1	1	1	1	1
**Total Content Data**	10	8	8	9	10
19. Date of issue or revision	1	1	1	1	1
20. Logo of issuing body	0	0	0	0	0
21. Producers of the document	0	0	0	0	0
22. Financiers of the document	0	0	0	0	0
23. Short bibliography of evidence-based data	1	1	1	1	1
24. Statement about patient involvement	1	1	1	1	1
**Total Identification Data**	3	3	3	3	3
25. Everyday language / explanation of jargon	0	1	1	0	1
26. Generic names for medications/products	N/A	0	1	0	1
27. Use of short sentences	1	1	1	1	1
28. Personal address to reader	0	0	0	0	0
29. Respectful tone	1	1	1	1	1
30. Clear information	0	0	0	0	0
31. Balanced risks and benefits	1	1	1	1	1
32. Logical presentation order	0	0	1	1	0
33. Satisfactory design/layout	1	1	1	1	1
34. Clear/relevant figures or graphs	N/A	1	N/A	N/A	N/A
35. Space for reader’s notes/questions	1	1	1	1	1
36. Printed consent form (contrary to recs)	N/A	N/A	N/A	N/A	N/A
**Total Structure Data**	5	7	8	6	7

**Table notes:** 1 = accurate and comprehensive; 0 = inaccurate, incomplete, or inconsistent; N/A = non-applicable; 0* = AI hallucinations.

Regarding the content, ChatGPT showed impressive capabilities in explaining the medical facets of diseases, treatment objectives, effects on quality of life, warning indicators, and highlighting crucial points (Items 3, 4, 11, 14, and 18). Concerning the date of publication or revision, ChatGPT offered specific dates for updates or releases, along with citations from relevant, evidence-based data sources. In terms of structure, ChatGPT’s replies were succinct and straightforward, with an average sentence length of around 15 words. The conciseness was greatly valued by professionals as it improved both understanding and the effectiveness of communication. Consequently, Item 27 demonstrated its efficient use of succinct language. In terms of design logic, ChatGPT employed a "broad-narrow-broad" format, beginning with a concise overview, moving into an in-depth exploration, and wrapping up with a recap of the essential elements. The expert evaluators deemed this method appropriate, as it notably enhanced the clarity and understanding of the content. As a result, Item 33 was given a score of 1, reflecting the uniform positive feedback from the reviewers.

In the content section, Item 8 received no score due to the discrepancy between the quantitative data supplied by ChatGPT and the cited literature. In the publication or revision date section, Items 20, 21 and 22 were not assigned any points due to the lack of pertinent details from ChatGPT. In the structural data segment, Items 28 was also left unscored for the same rationale. Furthermore, Item 30 did not receive a score because of incorrect details about surgical procedures, expenses, and quantitative benefits.

Of note, AI hallucinations occurred in all responses to Item 15 (regarding costs in five conditions), representing an occurrence rate of 2.8% (5/180).

**Fig 1:**
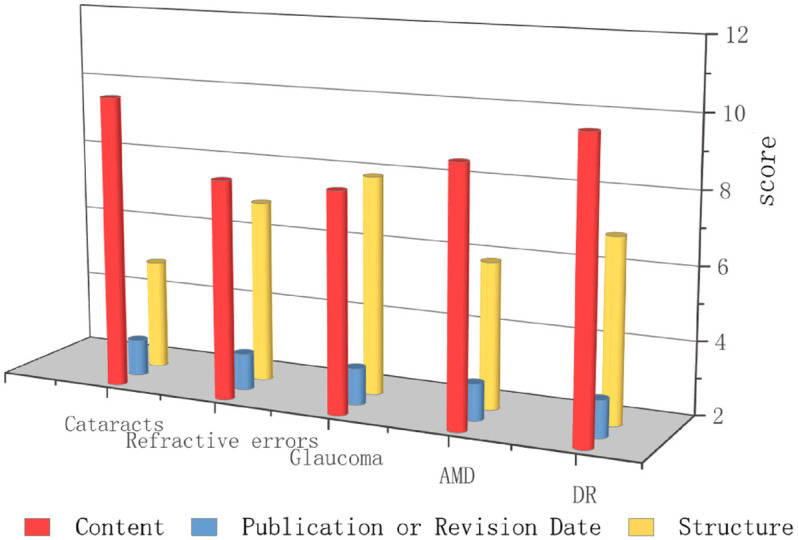
Comparison of scores across different diseases.

Overall, ChatGPT demonstrated the highest performance in providing medical information related to DR, with an EQIP total score of 20, ranking first among the five diseases. This was followed by glaucoma, which received a score of 19. In comparison, the model performed equally well for cataracts, refractive error, and AMD, each achieving a total score of 18. In the subdomain evaluation, in terms of content, both cataracts and DR received the highest score of 10. In the publication/revision date dimension, all conditions received identical scores of 3. Regarding structure, glaucoma achieved the highest score of 8. Detailed scoring results are presented in Fig 1.

The agreement between the two assessors, when evaluating ChatGPT’s responses with the adapted EQIP instrument, was quantified by a Cohen’s kappa value of 0.926 (95% CI 0.875-0.977, P < 0.001).

### 3.2 Conformity of ChatGPT’s responses to guideline suggestions

Across the five guidelines examined, the median score for ChatGPT’s replies was 4 (IQR 4-4), showing that its responses aligned with the suggestions from the analyzed guidelines. The consistency between the produced answers and the guideline recommendations was 84% (21/25), with a Cohen’s kappa value of 0.658 (95% CI 0.317-0.999, P < 0.001).

## 4 Discussion

This research thoroughly evaluated the accuracy of medical data produced by ChatGPT for five prevalent ophthalmic conditions (cataracts, refractive errors, glaucoma, AMD, and DR) by employing an advanced EQIP instrument and aligning with expert guidelines. The findings showed that ChatGPT generally excelled in content quality, especially regarding the precision and comprehensiveness of details about disease explanations, treatment objectives, and the effects on quality of life. However, major flaws were found in the organized display and clarity of information origins. For instance, it did not adequately specify the originating bodies, the identities of the participants, and the financial backers [17]. Moreover, ChatGPT experienced significant hurdles when tackling intricate problems like measuring quantifying benefits and risks or the expenses associated with medical treatments [18]. In the alignment assessment, ChatGPT’s replies aligned with 84% of the professional standards’ suggestions, demonstrating a high degree of accuracy consistent with specific clinical metrics [19].

The evaluation of ChatGPT’s responses for the five ophthalmic conditions highlighted various deficiencies, mainly in these aspects: (1) Ambiguous intervention steps and applicability criteria, particularly for conservative treatments of cataracts, refractive errors, and AMD (Item 5), ChatGPT did not adequately define the boundaries of non-surgical management, neglecting the personalized treatment requirements for these conditions. For example, early-stage cataracts or dry AMD may be addressed with conservative methods, while wet AMD necessitates pharmaceutical or surgical treatments [20]. The extent of non-invasive approaches for managing refractive issues was not clearly defined, potentially causing confusion about what patients should anticipate from such treatments. For the management of glaucoma and cataracts (Item 6), ChatGPT failed to outline detailed phase-specific strategies, which might result in patients having an unclear grasp of their medical situation [21,22]. (2) Insufficient data support, when addressing the quantification of risks and benefits (Item8, 10), ChatGPT did not provide specific clinical data or metrics, resulting in responses lacking scientific evidence and making inadequate data backing. This led to responses that lacked empirical support, hindering a thorough patient understanding of potential treatment outcomes and associated risks. (3) In discussing the qualitative benefits for glaucoma and AMD (Item 7), ChatGPT neglected to address the constraints of real treatment effectiveness, especially the impact of anti-VEGF therapy in AMD, which is primarily confined to decelerating the advancement of the disease. This oversight might result in patients harboring excessively optimistic expectations about the outcomes of their treatments. (4) Regarding refractive errors (Item 9), ChatGPT did not adequately outline the possible postoperative complications and their probabilities [23,24]. In summary, the constraints of ChatGPT in producing medical content highlight difficulties in managing intricate clinical situations and personalized healthcare requirements [25,26]. It is advisable to enhance and refine answers regarding intervention sequences and the management of patient expectations by incorporating expert medical advice [27,28]. In the future, the growing reliance on data-driven insights and personalized medical advice will bolster the precision and scientific foundation of ChatGPT’s responses, thereby boosting patient confidence and the caliber of healthcare decisions [29].

An examination of ChatGPT’s medical data uncovered cases of what is commonly referred to as "AI hallucination." This phenomenon occurs when the AI generates information that appears plausible but is actually baseless or deceptive [30]. For questions about the expenses related to the five conditions (Item 15), ChatGPT gave ambiguous answers like "it varies based on the situation" or "it might be beneficial," without providing concrete cost estimates or relevant influencing factors. Despite seeming thorough, these replies were not grounded in practical data and did not offer patients useful guidance. The problem of "AI hallucinations" underscores major shortcomings in AI-produced medical advice. While AI can generate responses that are structurally sound, its answers frequently lack scientific accuracy and real-world applicability, especially in scenarios demanding clinical expertise or exact data. This highlights the necessity for robust verification processes when using AI-generated health information, to avoid patients making incorrect health choices based on inaccurate data [31].

## 5 Conclusion

While ChatGPT, a swiftly advancing language model, shows initial promise in producing medical content, it also faces notable constraints. Currently, ChatGPT is still in the experimental phase, and its responses can be inaccurate or lack scientific rigor in some complex situations. Nonetheless, as technology progresses and its range of uses broadens, the model is expected to assume additional functions. These could include helping medical practitioners swiftly retrieve health data, aiding in patient education and wellness guidance, and acting as a supplementary instrument for analyzing medical research [32]. Future advancements ought to concentrate on boosting the model’s learning capacities and its access to scientific information, enhancing its dependability and practicality in medical settings, and catering to the individual requirements of patients and healthcare professionals.

## Supporting information

S1 File Published and repeat results for the Table 1 of the original experiments. (XLS)
